# Measurement of persistence in YAG:Ce^3+^ scintillator with pulsed synchrotron X-rays

**DOI:** 10.1107/S0909049511010843

**Published:** 2011-05-11

**Authors:** Tatsuhito Matsuo, Naoto Yagi

**Affiliations:** a Japan Synchrotron Research Institute, SPring-8, 1-1-1 Kouto, Sayo, Hyogo 688-5198, Japan; b Japan Science and Technology Agency, Kawaguchi, Saitama 332-0012, Japan

**Keywords:** high-speed camera, scintillator, synchrotron bunches

## Abstract

The decay time of YAG:Ce^3+^ phosphor was studied using a CMOS camera with a frame rate of 1302000 s^−1^ and pulsed X-rays from SPring-8. A high-resolution X-ray detector with YAG:Ce^3+^ was used with the camera to view the focused beam from the helical undulator. Mismatch between the ring circulation time and the frame time gave rise to a periodic variation of beam intensity in successive frames. Analysis of data obtained with two bunch modes showed that the decay time of YAG:Ce^3+^ was 60 ns. The variation of the beam positions in isolated bunches was small enough to be neglected in experiments using the focused beam. The results also show the possibility of an X-ray diffraction study at high time and space resolution.

## Abstract 2

The decay time of YAG:Ce^3+^ scintillator was estimated to be 60 ns by using a fast CMOS camera and synchrotron bunches.

## 1. Introduction

In experiments using synchrotron radiation, phosphor scintillators are widely used to detect X-rays (Martin & Koch, 2006 ▶; Gruner *et al.*, 2002 ▶). For a simple flux measurement, scintillation light from the phosphor can be detected by either a photomultiplier or a photodiode. For image acquisition, various types of camera can be used to view the scintillation. In these X-ray detectors one major concern is persistence of the scintillation. Although persistence does not affect intensity monitoring of constant X-ray flux, time-resolved measurements can be hampered by persistence. For this reason there have been many studies on the decay time of various phosphor scintillators. However, most of these were made on scintillation caused by electrons, not by X-rays, because the phosphors are more often used to convert electrons to visible light.

Among various phosphor scintillators, GADOX (Gd_2_O_2_S:Tb^+^, also known as P43 or GOS) is used most commonly. Although the efficiency and the photon stopping power of GADOX is among the highest of all phosphors, it has a persistence of 1–2 ms (Yagi *et al.*, 2004 ▶). The YAG:Ce^3+^ phosphor (Y_3_Al_5_O_12_:Ce^3+^, also known as P46) has a lower density than GADOX but is known to have much shorter persistence (Blasse & Bril, 1967 ▶) and thus has been used in high-speed X-ray detectors. In a previous study (Yagi *et al.*, 2004 ▶) the persistence was too short to measure accurately.

In the present study we used a high-speed CMOS camera and pulsed X-rays from synchrotron bunches to measure the decay time and weak afterglow of scintillation from YAG:Ce^3+^. We also examined the positional variance of the X-ray beam in isolated bunches.

## 2. Methods

A focused X-ray beam at the BL40XU high-flux beamline in SPring-8 was used. X-rays were produced by a helical undulator and used without monochromatization (Hara *et al.*, 2001 ▶). The beam was focused with two water-cooled bent mirrors. The coating of the first horizontally focusing mirror was Rh and that of the second vertically focusing mirror was Ni. The undulator gap was adjusted to obtain a peak X-ray energy of 15 keV. The X-ray beam was focused on the phosphor.

**Figure 1:**
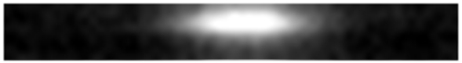
An X-ray beam recorded by the YAG:Ce^3+^ scintillator and CMOS camera. The image size is 64 by 8 pixels. The pixel is a 11.8 µm square at the phosphor. The X-ray beam size (FWHM) is about 150 µm horizontally and 40 µm vertically.

The X-ray detector was Beam Monitor 2 (AA50, Hamamatsu Photonics KK, Hamamatsu, Japan) (Uesugi *et al.*, 1999 ▶) with a *f* = 50 mm lens (Nikkor F# = 1.4, Nikon, Tokyo, Japan). The scintillator was a 20 µm-thick YAG:Ce^3+^ powder. The light from the scintillator was observed using a SA5 fast CMOS camera (Photron, Tokyo, Japan) equipped with a *f* = 85 mm lens (Nikkor F# = 1.4, Nikon). The two lenses worked together as a tandem lens. The pixel size at the phosphor was 11.8 µm × 11.8 µm. The SA5 CMOS camera works at 7500 frames s^−1^ with its full 1024 × 1000 pixels, but in the present study the area of view was restricted to 64 (horizontal) by 8 (vertical) pixels to obtain a frame rate of 1302000 s^−1^ (768 ns per frame). The camera has a 12-bit analog-to-digital converter. The focused X-ray beam was about 150 µm (horizontal) by 40 µm (vertical) full width at half-maximum. Thus, most of the beam fitted into the field of view (Fig. 1 ▶). The camera works in a global shutter mode so that there is no time delay within each image. During readout the camera was insensitive to light for about 230 ns in each frame (dead-time given by the manufacturer).

**Figure 2:**
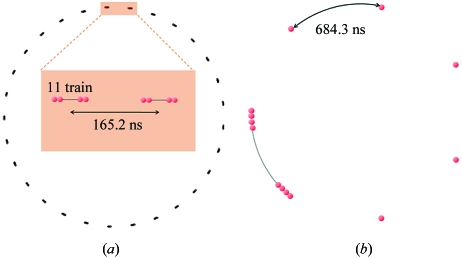
Filling mode of electrons at SPring-8. (*a*) ‘29 11-bunch trains’ mode (‘C mode’). (*b*) ‘1/7-filling plus 5 bunches’ mode (‘D mode’).

**Figure 3:**
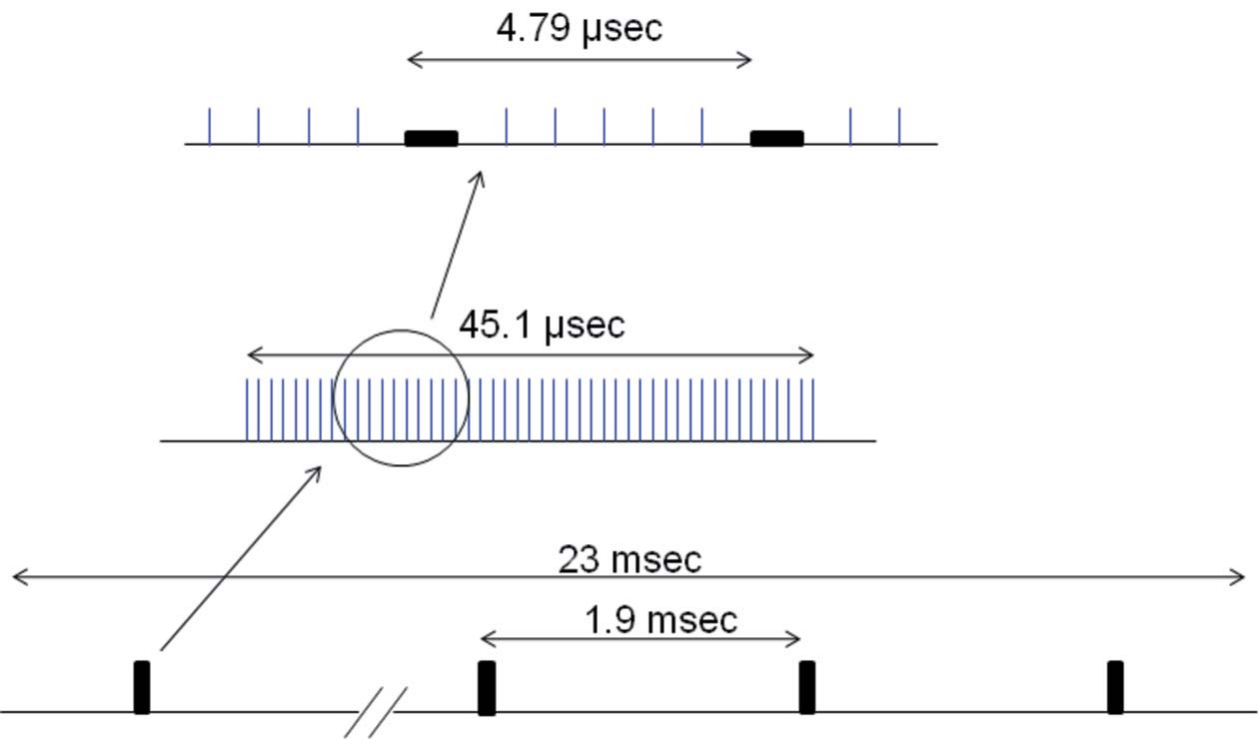
Shutter operation and bunch structure in the present experiment in the D mode. The recording by the CMOS camera was for 23 ms within which the rotating-disc shutter was open every 1.9 ms for 45.1 µs. In the 4.79 µs circulation time there is a 684.3 ns period with continuous bunches and five isolated single bunches.

Experiments were performed under two operation modes of the SPring-8 storage ring. One mode was the ‘29 11-bunch trains’ mode (2010A ‘C mode’, Fig. 2*a* ▶) in which 11 successive bunches (separated by 1.966 ns) formed one train and 29 trains were distributed evenly within the orbit. At the time resolution of the current study, 29 X-ray pulses were observed in one circulation. The periodicity of the trains was 165.2 ns and the gap between the trains was 145.5 ns. The other mode was the ‘1/7-filling plus 5 bunches’ mode (2010A ‘D mode’, Figs. 2*b* and Fig. 3 ▶) in which one-seventh of the orbit was occupied by full bunches with a total current of 85 mA, and five isolated bunches, each with 3.0 mA, were distributed evenly in the rest of the orbit. In this case the separation between the isolated bunches was 684.3 ns. We observed one long intense X-ray pulse and five small pulses in one circulation. Without an attenuator the flux at the detector was about 9 × 10^7^ photons per isolated bunch that was approximately 40 ps long. In both modes the circulation time of the SPring-8 storage ring was 4.79 µs and the total storage current was maintained at 100 mA by a top-up operation (Tanaka *et al.*, 2006 ▶).

The BL40XU beamline is equipped with a rotating disc with two symmetrically arranged slits that works as an X-ray chopper (Oka *et al.*, 2004 ▶). A slit height of 3.4 mm was chosen so that with a rotation of 16000 r.p.m. the X-rays were passed for 45.1 µs with 1.9 ms intervals (Fig. 3 ▶). This reduced the duty ratio to 2.4%. A slower X-ray shutter driven by a solenoid was used to further restrict the exposure time. These precautions were necessary to avoid radiation damage on the phosphor. In the present experiment, 30000 frames (23 ms) were recorded continuously. Thus, each recording contained 11–12 events of the 45.1 µs opening. To avoid saturation of the detector, an aluminium attenuator of 0.5–3 mm thickness was used.

For the study of the decay time the intensity in the whole area of each frame was integrated after dark subtraction. The center of the beam was estimated by calculating the center of gravity in the beam profile obtained by horizontal or vertical integration of the beam intensity.

## 3. Results

**Figure 4:**
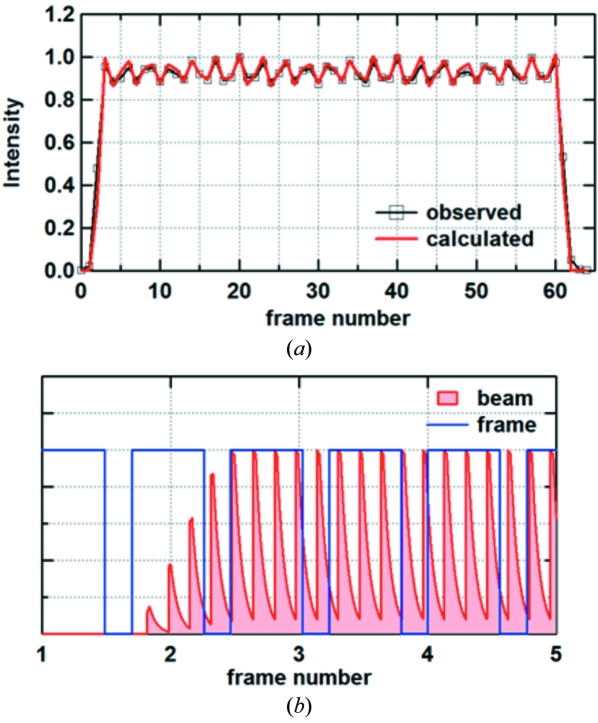
Intensity fluctuation observed in the C mode. (*a*) A typical intensity fluctuation observed with the CMOS camera. Squares are the integrated beam intensity and the red curves are the simulated intensity based on a decay time constant of 60 ns and dead-time of 210 ns. At the beginning and the end of the 45 µs opening the edges of the slit in the rotating disc move across the X-ray beam, making frames with intermediate intensity. (*b*) Schematic diagram showing how the intensity fluctuation is created.

In the C mode the integrated intensity within the 45 µs period fluctuated by about 10% peak-to-bottom (Fig. 4 ▶). On average, there was an approximate three-frame repeat with rather irregular intensities. This is due to a mismatch (aliasing) between the train frequency (1/145.5 ns) and the frame rate (1/768 ns) which created frames containing either four or five trains. Computer calculation was performed to simulate the observed intensity fluctuation assuming an exponential decay of the YAG:Ce^3+^ fluorescence (Fig. 4 ▶). Generally, the simulated intensity matched the observation with a scintillation decay time constant of 60 ns and a camera dead-time of 210 ns. An equally good fit was obtained with a time constant of 57–60 ns and dead-time of 209–211 ns. The decay time is similar to that previously reported for excitation by electrons (70–80 ns) (Blasse & Bril, 1967 ▶). It is notable that, even though the persistence from the pulses is gradually summed up, the intensity settled at a certain level. If the persistence from the previous pulses decays with exp(−*t*/τ), where *t* is the time after the pulse and τ is the decay time constant, it becomes the sum of a geometric series, which approaches asymptotically a constant value. The result shows that there is not significant persistence with a longer time constant.

**Figure 5:**
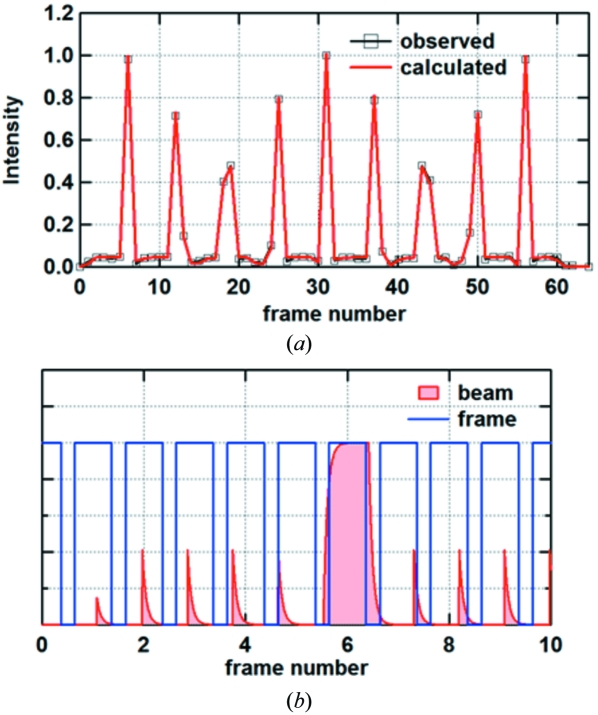
Intensity fluctuation observed in the D mode. (*a*) A typical intensity fluctuation. Squares are the integrated beam intensity and the red curves are the simulated intensity based on a decay time constant of 60 ns and dead-time of 210 ns. (*b*) Schematic diagram showing how the intensity fluctuation is created.

In the D mode there were frames with high, low and very low intensity (Fig. 5 ▶). The frames with high intensity corresponded to the 1/7 filled portion in the storage ring (Fig. 2*b* ▶). The frames with low intensity contained one isolated bunch. The frames with very low intensity did not contain any bunch but the intensity is not zero because of the persistence from the bunch before the frame. Again, computer simulation showed that a decay time of 60 ns and dead-time of 210 ns can account for the observed intensity fluctuation.

The differences in the beam positions of the one train and the five bunches were less than 1 µm horizontally and 0.1 µm vertically. These results show that the five isolated bunches have the same beam profile and do not increase the apparent beam size observed by longer exposure.

## 4. Discussion

Persistence is an important problem in time-resolved X-ray experiments. In time-resolved diffraction or imaging experiments CCD and CMOS cameras are used most often. Since these cameras are prone to damage caused by hard X-ray photons and the sensors are often too small for the required area size, they are used with a phosphor scintillator and an appropriate coupling like lens- or fiber-coupling. With the advent in CMOS technology, the framing rate of CMOS cameras now exceeds the decay time of the GADOX phosphor that is used most commonly. Thus, the choice of phosphor for the CMOS cameras is now a serious concern.

The fluorescence intensity of YAG:Ce^3+^ is about 40% of that of GADOX. However, the present results show that it can be used in experiments in the submicrosecond range. In the present study we demonstrated that the bunch structure of the SPring-8 storage ring can be observed and positions of the X-ray beams given by the isolated bunches can be examined with this phosphor. One important advantage of YAG:Ce^3+^ is that it can be used as a crystal scintillator to obtain spatial resolution better than 1 µm (Martin & Koch, 2006 ▶), making it possible to achieve both very high time and spatial resolution.

One of our aims in future is to make an ideal detector for the single-molecule tracking experiments (Sasaki *et al.*, 2001 ▶; Shimizu *et al.*, 2008 ▶). This technique makes use of white Laue diffraction from a gold nanocrystal that is attached to a protein molecule. By observing the trajectory of a diffraction spot one can study the rotational motion of the protein molecule. Since the motion of a protein in solution or in a membrane is fast, measurements on a microsecond range are required. To follow diffraction patterns, the detector may need to have a larger area, like an X-ray image intensifier (Amemiya *et al.*, 1995 ▶). A detector with YAG:Ce^3+^ is also useful for X-ray photon correlation spectroscopy experiments (Shinohara *et al.*, 2007 ▶) which require high time and spatial resolution. For time-resolved X-ray diffraction experiments in general, the present results demonstrate the possibility that the intensity and position of a diffraction spot can be measured at submicrosecond time resolution with high spatial accuracy.

An X-ray free-electron laser (XFEL) produces X-rays in very short pulses. The pulse intervals are different in the XFEL facilities that are currently operational or under construction. At the Linac Coherent Light Source (LCLS) in the USA and the SPring-8 Compact SASE Source (SCSS) in Japan, the maximum repetition rate is 120 Hz (currently 60 Hz) and 60 Hz, respectively. At the European XFEL in Hamburg the pulses are separated by only 200 ns (these pulses are delivered in bunch trains, 0.6 ms long, containing 3000 bunches, followed by 99.4 ms gaps). In experiments using these facilities it is required to record a diffraction or scattering pattern produced by each pulse. With a pulse rate of 60 or 120 Hz, this is possible with currently available CCD or CMOS cameras, as demonstrated in the present study. One concern in such an experiment is the persistence in the phosphor scintillator. Even though the images can be read out between the pulses, high time resolution cannot be achieved if the image from the previous pulse overlaps because of persistence. The present results show that the decay time of YAG:Ce^3+^ is short enough for such experiments. The repetition interval at the European XFEL is shorter than at other facilities but still longer than the decay time of YAG:Ce^3+^. The frame rate of the CMOS camera used in the current study is not fast enough but it is conceivable that the CMOS technology will eventually develop to acquire an image with each pulse.
